# Unlocking the Power of Artificial Intelligence: Accurate Zeta Potential Prediction Using Machine Learning

**DOI:** 10.3390/nano13071209

**Published:** 2023-03-29

**Authors:** Rizwan Muneer, Muhammad Rehan Hashmet, Peyman Pourafshary, Mariam Shakeel

**Affiliations:** 1 School of Mining and Geosciences, Nazarbayev University, Astana 010000, Kazakhstan; 2 Department of Chemical and Petroleum Engineering, United Arab Emirates University, Al Ain 15551, United Arab Emirates

**Keywords:** zeta potential, nanoparticles, nanofluids, colloidal system, artificial neural networks

## Abstract

Nanoparticles have gained significance in modern science due to their unique characteristics and diverse applications in various fields. Zeta potential is critical in assessing the stability of nanofluids and colloidal systems but measuring it can be time-consuming and challenging. The current research proposes the use of cutting-edge machine learning techniques, including multiple regression analyses (MRAs), support vector machines (SVM), and artificial neural networks (ANNs), to simulate the zeta potential of silica nanofluids and colloidal systems, while accounting for affecting parameters such as nanoparticle size, concentration, pH, temperature, brine salinity, monovalent ion type, and the presence of sand, limestone, or nano-sized fine particles. Zeta potential data from different literature sources were used to develop and train the models using machine learning techniques. Performance indicators were employed to evaluate the models’ predictive capabilities. The correlation coefficient (r) for the ANN, SVM, and MRA models was found to be 0.982, 0.997, and 0.68, respectively. The mean absolute percentage error for the ANN model was 5%, whereas, for the MRA and SVM models, it was greater than 25%. ANN models were more accurate than SVM and MRA models at predicting zeta potential, and the trained ANN model achieved an accuracy of over 97% in zeta potential predictions. ANN models are more accurate and faster at predicting zeta potential than conventional methods. The model developed in this research is the first ever to predict the zeta potential of silica nanofluids, dispersed kaolinite, sand–brine system, and coal dispersions considering several influencing parameters. This approach eliminates the need for time-consuming experimentation and provides a highly accurate and rapid prediction method with broad applications across different fields.

## 1. Introduction

Nanoparticles have gained significant importance in science and technology due to their unique properties and potential applications. Nanoparticles exhibit increased reactivity and altered material properties due to their high surface-area-to-volume ratio. This can lead to more efficient chemical, biological, and catalytic reactions as well as improved strength, toughness, and conductivity of materials [1]. Nanoparticles have shown great potential in medical applications such as drug delivery, imaging, and cancer therapy, as they can be designed to target specific cells and tissues [2,3,4,5]. In addition, they are being explored for their use in the oil and gas industry [6,7,8,9,10,11,12,13,14,15], energy storage and conversion [16,17,18,19], environmental remediation [20,21], and information and communication technology, including the manufacture of smaller and faster electronic devices and data storage systems [22,23]. Overall, nanoparticles hold great potential for a wide range of applications and their unique properties make them a promising area of research.

Nanofluids are advanced types of fluids that contain dispersed nanoparticles and a base fluid such as water, oil, or ethylene glycol. These nanoparticles, which are typically less than 100 nanometers in diameter and made of materials such as metals, oxides, and carbides, modify the fluid’s properties and improve its performance. To create nanofluids, the nanoparticles are first synthesized through techniques such as chemical precipitation or sol-gel synthesis. They are then dispersed into the base fluid using methods such as ultrasonication, magnetic stirring, or high-pressure homogenization. To ensure the stability of the nanofluids, techniques such as adding surfactants or electrostatic stabilization are used [24]. During nanofluid preparation, it is required to prevent the aggregation of nanoparticles and maintain a stable dispersion to obtain maximum efficiency. On the other hand, coagulation may also be advantageous in certain industries, such as water treatment or water filtration. This rapidly growing field of research offers a wide range of applications due to their unique properties and the improved performance of nanofluids when compared to traditional fluids. Nanoparticles, which can be made from metal oxides, metals, or carbon-based materials, give the fluid new and distinct thermal, electrical, and optical properties [25,26]. The advantages of nanofluids include enhanced oil recovery from petroleum reservoirs [27,28], fines migration control in sandstones [10,29,30], enhanced thermal stability [31,32], corrosion resistance [33,34], and potential uses in other fields such as the aerospace, automotive, and textile industries [35,36,37,38,39,40,41].

Zeta potential is a measure of the electrical charge that exists on the surface of nanoparticles present in a dispersion. The electrical charge on the surface of particles in the colloid arises from the adsorption of ions from the surrounding medium [10,42,43]. The electrical charge is produced on the surface as a consequence of local free electrons in the solution. These local free electrons tend to rearrange themselves into a non-zero charged zone that resides close to the particle–solution interface. An electric double layer (EDL), comprising a compact layer and a diffuse layer, is developed due to the distribution of charges at solid–liquid interface and the equilibrium between the positive–negative ions in the liquid. It is a thin, tightly packed layer of ions that is present just adjacent to the surface of a charged particle. Since there is a high electrostatic attractive force present, the ions in this layer remain immobile [44,45]. On the other hand, beyond this layer, the ions in the solution are free to move about. The electrostatic potential at the border separating the compact layer from the diffusion layer is called the zeta potential.

Zeta potential can be positive or negative and plays a key role in determining the stability of nanofluids and colloidal systems [46,47]. A high absolute value of zeta potential indicates a more stable and dispersed nanofluid. Conversely, a low absolute value of zeta potential signals a greater chance of the particles settling out or aggregating [48]. Dispersions containing nanoparticles are among the key situations in which zeta potential measurement is quite helpful. Among various types of nanoparticles, silica, titanium dioxide, carbon nanotubes, copper, clay, and aluminum oxide are the most extensively utilized nanoparticles in the oil and gas and energy sector, pharmaceuticals, the automobile industry, the construction sector, and textile and paint sectors [16,36,40,41,42]. Silica nanoparticles are widely utilized in the petroleum industry due to their advantageous combination of small size, high surface area, and versatility, making them effective at improving oil recovery, fines migration control, drilling fluid stability, and removing contaminants from crude oil [10,14,49,50,51,52,53,54,55].

The zeta potential of silica nanoparticles is influenced by various factors such as the surrounding pH, salt concentration, temperature, and surface modification. The negative zeta potential of silica nanoparticles is primarily due to the presence of silanol groups on their surface that can ionize to form negatively charged silicate species in an aqueous solution. In this context, a more negative zeta potential can prevent the aggregation and sedimentation of silica nanoparticles in a colloid, thus maintaining the stability of the system. Zeta potential is often used as a stability indicator for nanofluids and is considered an important factor in determining the quality and shelf life of these fluids. Therefore, it is essential to have an understanding of zeta potential since this is a critical parameter to take into account while preparing stable dispersions. 

The measurement of zeta potential is essential in a wide variety of industries, including the upstream petroleum sector to determine the interaction between oil, water, and rock [56,57,58,59,60,61,62,63]; the interaction between cement and surfactant, and cement hydration and adsorption in the construction industry [64,65]; wastewater treatment and the optimization of water quality [66,67]; pigment dyeing in the textile industry [68,69]; mineral extraction from suspensions and solvents [70,71]; and brewing yeast mixtures and beverage processing [72,73].

Several factors can influence the surface charge of nanoparticles and colloidal materials, which, in turn, affect the zeta potential [74,75]. The pH of the aqueous medium and the temperature of the dispersion [8,76,77,78,79], the ionic strength [8,9,10,42,76], the presence of monovalent and divalent ions and other substances such as polymers and surfactants in the solution [42,80,81], the weight percentage of the solute in the dispersion [8,10,42], and the utilization of ultrasonication [80] are other critical parameters that influence the surface charge of the colloidal system

Quantification of the zeta potential is one of the most straightforward techniques for describing the surface charge of the particles in colloidal systems. Knowing the zeta potential value as well as the variables that influence it enables us to carefully evaluate the stability of colloidal systems. To determine the zeta potential, the electrophoretic mobility is measured and then used to calculate the zeta potential through the Smoluchowski equation, as shown in Equation (1) [82].

(1) $$\zeta =\frac{3\;U_{E}\;\eta }{2\;\varepsilon \;F\left(ka\right)}$$

The equation takes into account various factors such as particle speed, fluid viscosity, electric field strength, and the balance between the electrical repulsion and friction forces in the fluid. This equation is widely used in the field of colloid science to quantify zeta potential and is a valuable tool for comprehending the charge on particles in fluids. The streaming potential method is another commonly used technique to determine the zeta potential of colloidal materials. The magnitude of the streaming current is proportional to the zeta potential of the particles in the solution [83,84]. The zeta potential may also be predicted with the use of modeling techniques, such as molecular simulations [74] and a Monte Carlo simulation [85]. There have been few studies on the prediction of zeta potential using one machine learning technique such as ZPRED or neural networks. In addition, these studies have only considered a single medium with limited variables [86,87]. The current research proposes various machine learning approaches considering several mediums and variables to at least partly substitute mathematical modeling for experimentation. In artificial neural networks, the factors that have the largest effect on the value of the zeta potential are typically employed as inputs. This is because these variables can best predict the future values of zeta potential.

Since the early 1990s, researchers in the natural sciences have started making use of artificial neural networks [88,89,90]. They are composed of interconnected artificial neurons, arranged in layers. The input layer receives data which is processed by the hidden layers and the output layer delivers the computed results. Each artificial neuron in an ANN performs basic mathematical operations on the inputs received and outputs a value that is then transmitted to the next layer. Artificial neural networks are employed by the petroleum industry to forecast reservoir performance, polymer viscosity, stability, particle size, well-log interpretation, and enhanced oil recovery prediction [91,92,93,94,95,96].

Considering the effect of all the influencing factors, zeta potential measurement is a time-consuming process that calls for an in-depth understanding of the experimentation process and resource utilization. This research aimed to develop zeta potential prediction models using data collected from previous experiments, employing artificial neural networks (ANNs), support vector machines (SVMs), and multiple regression (MRAs) analyses, and to compare the accuracy of these three methods. In addition, zeta potential predictions were made using the most accurate ANN model, and the results were encouraging. The ANN model developed in this study is the first-ever model to predict zeta potential based on various influencing parameters, offering broad applications across multiple fields. The proposed method eliminates the need for lengthy experimentation and provides a rapid and accurate prediction approach while taking into account several critical parameters that can impact zeta potential.

## 2. Research Methodology

### 2.1. Data Collection

To have a comprehensive data set, zeta potential data were obtained from published sources, collecting 249 data points. Zeta potential data were collected, considering critical parameters such as ionic strength, pH, temperature, presence of monovalent ions, type and concentration of silica nanoparticles, and medium. The zeta potential data were based on silica nanoparticles, covering a size range of 10–50 nm. The measurements were taken across a wide range of experimental conditions, including varying concentrations of silica nanoparticles, ranging from 0.005–6 wt%; temperatures, ranging from 20–150 °C; and salinities for NaCl (30–315,000 ppm) and KCl (500–60,000 ppm). Furthermore, the pH range of the samples extended from 1 to 11, thereby offering insight into the zeta potential behavior under acidic, neutral, and alkaline conditions. Furthermore, the zeta potential data were collected for five different mediums, including water, sand, glass beads, coal, and kaolinite. These mediums were selected to investigate the effect of different surfaces on the zeta potential. In order to model the effect of the medium, which is a categorical variable, a few preprocessing steps were followed to transform it into a numerical variable. The one-hot encoding method was used which involved converting each category of the categorical variable into a binary variable. For instance, since our categorical variable had five categories, then five binary variables were created, where each variable took on value 1 if the category was present and 0 otherwise. The collected data are presented briefly in Table 1. The complete dataset is available as a Supplementary Data file for further reference. In addition, in our previous study, we measured the zeta potentials of sand dispersions containing silica nanoparticles and used them to validate the predictions of our model [10].

### 2.2. Data Analysis Techniques

The primary objective of this work was to develop a model that can accurately predict the zeta potential of dispersion, while considering influencing factors, without requiring extensive laboratory experiments. To achieve this goal, three machine learning approaches were utilized, as follows:

#### 2.2.1. Multiple Linear Regression (MLR)

Multiple linear regression is a statistical technique used to examine the relationship between an outcome or dependent variable and several predictor or independent variables by establishing a linear equation based on observed data. It presumes a straight-line relationship between the dependent and independent variables and aims to reduce the deviations or differences between the observed and estimated values of the dependent variable. The objective of MLR is to identify the optimal coefficients for the predictor variables that produce the lowest deviations and the most accurate prediction of the outcome variable. The fitlm function in MATLAB is used to analyze the relationship between different variables in MLR. Outliers are then identified and removed. By utilizing the least-squares method, this function produces a linear regression model where one or more independent variables, or predictors, are combined linearly to form a dependent variable. Finally, the coefficients of terms are calculated. The plot function is used to visualize and interpret the results.

#### 2.2.2. Support Vector Machine (SVM) Model

Support vector machines (SVM) is an algorithm used for supervised machine learning that can handle both classification and regression problems. It works by finding the optimal boundary, called a hyperplane, that separates the data into different classes or predicts a continuous outcome by maximizing the margin between the closest data points, called support vectors, and the hyperplane. The SVM algorithm is capable of handling non-linearly separable data by transforming it into a higher dimensional space where a linear boundary can be established. This method is versatile and can be applied to a wide range of data types. The SVM method is based on kernel functions and thus it is a nonparametric method. The nonlinear SVM regression algorithm uses a nonlinear kernel function to map the inputs to a high-dimensional space. The model tries to obtain the coefficients that decrease the Lagrangian function. Different solver algorithms are available to solve the SVM optimization problem. In this study, the Gaussian or radial basis function ‘rbf’ was used as a kernel function while the sequential minimal optimization (SMO) technique was used to solve the regression optimization problem. A convergence criterion was specified, and the solver stopped computation once the criterion was met. 

The optimum SVM regression model is obtained by optimizing the hyperparameters. As a result of optimization, the regression output with the lowest calculated cross-validation loss is obtained. The model generalization is evaluated by computing the loss of the model on the test dataset. To evaluate the accuracy of the zeta potential predictions, performance parameters such as the root mean square error (RMSE), mean absolute percentage error (MAPE), and correlation coefficient (r) were calculated for both the training and testing datasets. These parameters were computed by comparing the actual and predicted values of the zeta potential.

#### 2.2.3. Artificial Neural Network (ANN) Model

In general, ANN models consist of input parameters, hidden layers with neurons, an activation function, and output layers interconnected through neurons. Signals from one layer are summed up in the next layer. The training process then applies the “bias and weights” procedure to this summed value. Each signal from neurons is estimated by an activation function. In order to effectively predict zeta potential using an ANN model, the input layer must contain variables that have an impact on zeta potential. Therefore, the selection of neurons for the input layer takes into account the behavior of these parameters and their effect on zeta potential measurement conditions. A training process consisting of 1000 epochs was conducted to ensure the model achieved optimal performance. There are several types of ANN models according to the structure and method of processing data. One of the most common ANN models is a feed-forward neural network where neurons of the input layer connect to one following layer. In this study, the ‘*trainbr*’ training function was employed where Levenberg–Marquardt optimization was used to update the weight and bias variables. To create a network that generalizes well, it first identifies the optimal mix of squared errors and weights. 

**Figure 1:**
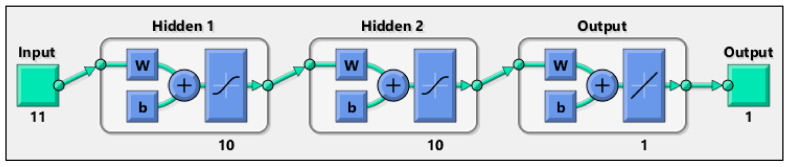
A schematic of the developed ANN model.

Data sets are converged to a matrix, imported into MATLAB script, and normalized. These parameters are used for training, validating, and testing the models. The data points are randomly divided into 2 sets including training (80%), and testing (20%), as evidenced by some recent studies [118,119,120]. The Levenberg–Marquardt method is used to train the feed-forward ANN model. The fitnet function in MATLAB is used to fit an ANN model to the data. Furthermore, the ‘trainbr’ training function is employed where Levenberg–Marquardt optimization is used to updating the weight and bias variables. To create a network that generalizes well, it first identifies the optimal mix of squared errors and weights. The trial-and-error process is carried out according to the hyperbolic tangent sigmoid transfer (tansig) activation function in the hidden layers and the pure linear transfer function (purelin) in the output layer as a suitable structure of the ANN model. In addition, the accuracy of the model directly depends on the number of hidden layers and the number of neurons. Various ANN model configurations were evaluated, and the final model was selected with two hidden layers having ten neurons in each layer, as schematically shown in Figure 1. The finalized model was run several times to obtain the best values of the correlation coefficient for the actual and predicted values for both data sets.

To evaluate the generalization of the models, three performance parameters were estimated using all the data analysis methods. The correlation coefficient (r), root mean squared error (RMSE), and mean absolute percentage error (MAPE) were calculated and compared to determine the best method for predicting zeta potential.

The correlation coefficient is an important statistical measure to evaluate the goodness of a model. It ranges from 0 to 1, where 0 indicates no relationship and 1 indicates a perfect agreement between the actual and predicted values. Equation (2) is used to calculate ‘*r*’ [121].

(2) $$r=\frac{\sum \left(x_{i}-\bar{x}\right)\left(y_{i}-\bar{y}\right)}{\sqrt{\sum {\left(x_{i}-\bar{x}\right)}^{2}\sum {\left(y_{i}-\bar{y}\right)}^{2}}}$$

*RMSE* is a statistical measure used to evaluate the generalization ability of a model. It is calculated as the square root of the mean of the squared differences between the predicted and actual values of the target variable. *RMSE* measures the average difference between the predicted and actual values and gives an idea of how much error the model makes in its predictions. A lower *RMSE* value indicates that the model has high accuracy, while a higher *RMSE* value indicates that the model is less accurate. The *RMSE* was calculated using Equation (3).

(3) $$RMSE=\sqrt{\frac{\sum_{i=1}^{n}{\left(\mu_{p}\left(i\right)-\mu_{a}\left(i\right)\right)}^{2}}{n}}$$

The mean absolute percentage error (*MAPE*), as given by Equation (4), is another important statistical measure for evaluating the quality of a model. A model is considered efficient and accurate if its *MAPE* is small or closer to zero.

(4) $$MAPE=\frac{1}{n}\overset{n}{\underset{i=1}{\sum }}\frac{\left|\mu_{p}\left(i\right)-\mu_{a}\left(i\right)\right|}{\mu_{a}\left(i\right)}$$

## 3. Results and Discussion

The machine learning techniques of MLR, SVM, and ANN were employed, and the results are presented in the following section.

### 3.1. The MLR Results

**Figure 2:**
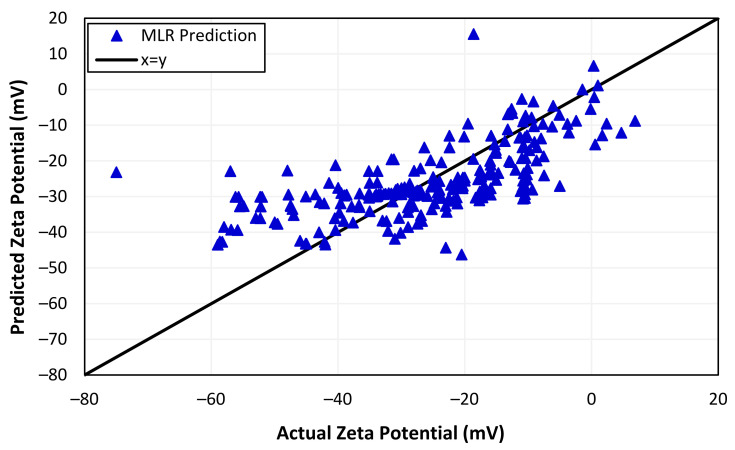
Actual and predicted zeta potentials from MLR.

The MLR outputs represented in Figure 2 show that the predicted zeta potentials by the multiple linear regression model do not match the actual values accurately, with a correlation coefficient of 0.68. It shows a positive correlation, but the relationship is not perfect. This could be due to the MLR model’s inability to capture the underlying nonlinearity of the data and the scarcity of available data points, limiting the generalization capability of the model. Moreover, Table 2 shows the other estimated indices for the predicted zeta potentials based on the MLR model. A high RMSE of 10.722 shows how much predicted values deviate from actual values of zeta potential using the approach of Euclidean distance. Similarly, a *MAPE* value of 0.265 indicates that the average deviation of the forecast from the actual value is 26.5%, meaning that the forecast values are off by roughly one-quarter of the actual values. This indicates that the inadequacy of the MLR model to predict zeta potential accurately could also be due to the model’s reliance on multiple predictors, leading to underfitting and, hence, poor generalization.

### 3.2. The SVM Results

**Figure 3:**
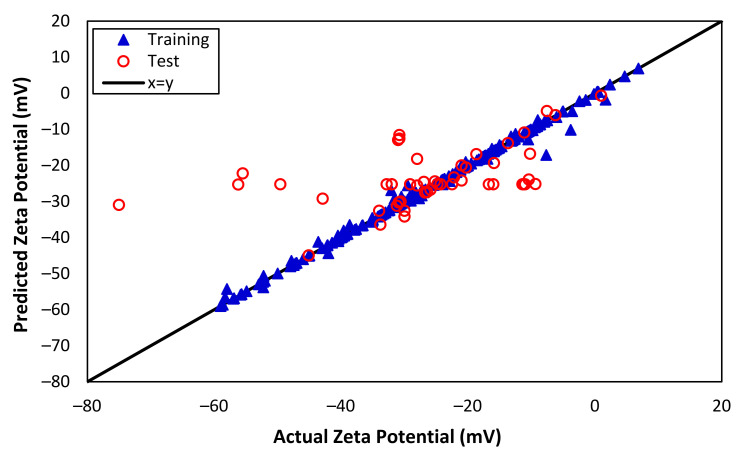
Actual and predicted zeta potentials from SVM (training and test data).

The results of the SVM model for zeta potential modeling and prediction are presented in Figure 3. It is evident from the figure that the SVM model performs substantially better than the MLR model, especially during ‘training’ as it considers the non-linear relationship between the response and predictors. Various kernel functions, such as linear, rbf, and polynomial, were tested, and the ‘rbf’ kernel function delivered the most accurate predictions of the zeta potential, with an excellent correlation coefficient of over 0.997 for the training set. This suggests that the SVM model has a stronger ability to capture the non-linear patterns in the data, leading to improved performance compared to the MLR model. However, the correlation coefficient of the ‘test’ data is 0.516, showing that the SVM model is unable to predict the zeta potential effectively due to the complexity of data with several variables. Table 3 provides a comprehensive summary of the estimated indices and correlation coefficients for the predicted zeta potentials based on the SVM model for both training and test data. The *RMSE* and *MAPE* for training data are 1.155 and 0.0191, respectively, indicating an excellent capturing of data points during training. On the other hand, the higher values of *RMSE* and *MAPE* of test data, 12.271 and 0.2881, respectively, show the inability of the SVM model for prediction.

#### Zeta Potential Prediction Using SVM Model

**Figure 4:**
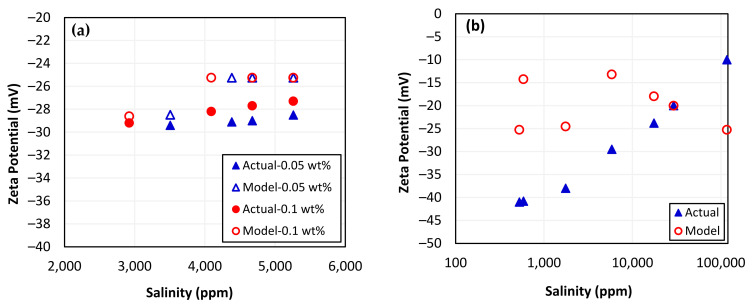
Zeta potential predictions using SVM model: (**a**) sand with nanoparticles and (**b**) only sand.

The measurement conditions for the zeta potential of the silica nanoparticles–sand–brine system were obtained from our previous study [10] and used as input for the SVM model to make predictions. To ensure consistency and reliability in the model’s outcomes, the operating conditions were kept the same. The results of zeta potential prediction in the presence of silica nanoparticles using the SVM model were not encouraging, as indicated in Figure 4a, with a correlation coefficient of only 0.60. Likewise, the zeta potential measurement conditions for the sand–brine system without silica nanoparticles were obtained from the literature [107] and used as input to predict the zeta potential, and the results are shown in Figure 4b. The low correlation coefficient of 0.15 indicates that the SVM model was unable to predict zeta potential when provided with the conditions of unseen data. Although the SVM model performed well during the training phase, it was unable to capture the complexity of the zeta potential data with multiple variables and provide satisfactory results. The SVM model failed in prediction due to overfitting (r = 0.997), whereas the model worked perfectly on training data but failed to generalize to new data. There have been studies that suggest that SVM models may not be able to accurately predict outcomes in complex datasets which contain multiple variables, are not linearly separable, or have outliers [122,123].

### 3.3. The ANN Results

**Figure 5:**
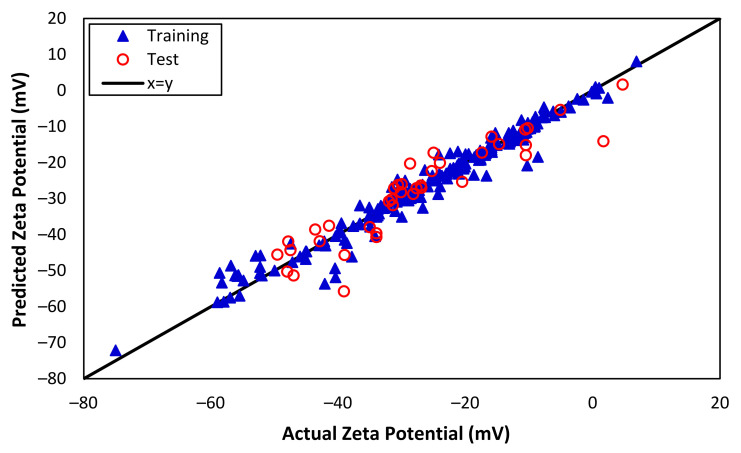
Actual and predicted zeta potentials from ANN (training and test data).

Figure 5 presents the impressive performance of the ANN model for predicting zeta potential, with predicted values closely matching the actual zeta potential. The correlation coefficients for training and test data are 0.982 and 0.923, respectively. When the correlation coefficient for the training data is 0.982 and for the test data is 0.923, it suggests that the ANN can capture the non-linearity of both the training and test data with a high degree of accuracy. This indicates that the model performs well at predicting the outcomes for new, unseen data, and is not just overfitting the training data. In addition, Table 4 provides a detailed overview of the model performance, including the indexes that suggest excellent agreement between the predicted and actual values. The RMSE values for training and test are 2.754 and 5.347, respectively, indicating that the model’s predictions are highly concentrated around the best-fit line. Additionally, MAPE for both training and test data are 0.053 and 0.131, respectively, indicating that the model performs exceptionally well on both training and test data. Overall, the ANN model demonstrated a significantly better performance than both the MLR and SVM approaches, which is a testament to its robustness and effectiveness in capturing the non-linear patterns and relationships within the data.

#### Zeta Potential Prediction Using ANN Model

**Figure 6:**
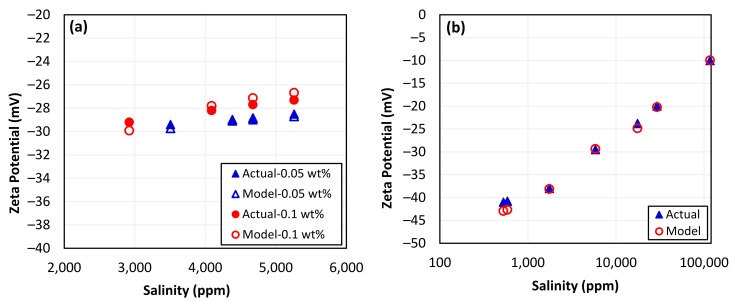
Zeta potential predictions using ANN model: (**a**) with nanoparticles and (**b**) with sand.

The most accurate predictions of zeta potential for systems involving silica nanoparticles–sand–brine and sand–brine were made using the ANN model that performed the best. The zeta potential measurement conditions used for the ANN model were obtained from our previous study [10] and kept consistent to ensure reliable and accurate predictions. As depicted in Figure 6a, the ANN model was able to generate zeta potential predictions that were in close agreement with the measured data. With a correlation coefficient of 0.972 for actual and predicted values, the developed ANN model performed remarkably well at predicting the zeta potential of the silica nanoparticles–sand–brine system with varying concentrations of nanoparticles and other influencing parameters. This high level of accuracy suggests that the ANN model was able to perform excellently even when presented with unseen data. Similarly, the zeta potential measurement conditions for the sand–brine system alone were also obtained from the literature and utilized as input to predict the zeta potential [107]. The ANN model was also able to accurately predict the zeta potential for a sand–brine system alone, with a remarkably strong correlation coefficient of 0.998 for actual and predicted values, as presented in Figure 6b. This indicates that the ANN model was highly effective at producing accurate and reliable predictions for this particular system as well. This approach helped to ensure that the predictions generated by the ANN model were based on reliable and consistent data, which would allow for accurate comparison with the actual experimental results.

### 3.4. Comparative Analysis of ANNs and SVM

**Figure 7:**
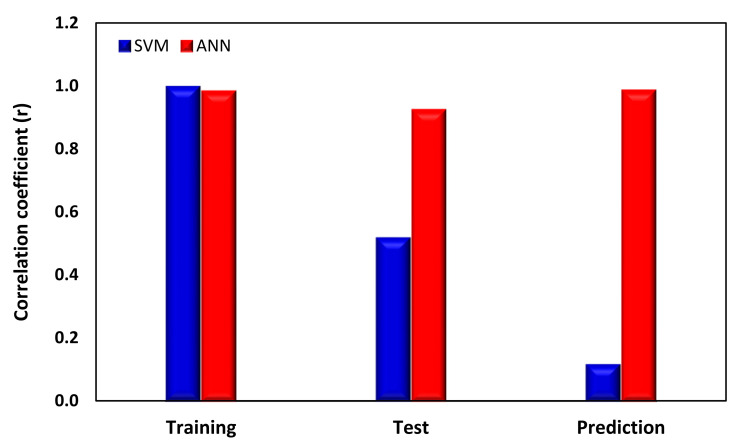
Comparative analysis of SVM and ANN models.

In this study, the performance of three distinct machine learning methods for predicting zeta potential based on seven input parameters was assessed and compared. The outcomes of these different techniques were analyzed and evaluated using various performance metrics, including correlation coefficient, MAPE, and RMSE values, as shown in Table 2, Table 3 and Table 4. These results indicate that the ANN model is the most effective approach for predicting zeta potential with multiple independent parameters, due to its ability to capture complex non-linear patterns within the data and produce highly accurate predictions, as presented in Figure 7. The SVM model, which also considers non-linearity, achieved excellent results for training data but failed with test data and prediction with unseen data. There could be various reasons why the ANN outperforms a support SVM on the test data, despite the SVM offering a superior correlation coefficient during training. Overfitting of the training data could have occurred, leading to a poor generalization of the SVM model. ANNs are more adept at generalizing to new data and can capture nonlinear relationships between variables, whereas SVMs may struggle to recognize the underlying pattern in the data. SVM performance relies heavily on kernel selection and associated parameters, which, if not optimized, can result in a subpar performance on test data. ANNs are more suited to data with complex nonlinear relationships, whereas SVMs excel on data with a clear boundary between classes. Overall, this study provides valuable insights into the effectiveness of different machine learning techniques for predicting zeta potential and highlights the importance of selecting appropriate methods that can effectively handle the complexity and non-linearity of the zeta potential data when dealing with various influential parameters.

## 4. Conclusions

Many sectors, such as pharmaceuticals, construction, pigment production, petroleum, mineral processing, and others, rely on the measurement of the zeta potential to assess the stability of colloidal suspensions. Therefore, the prediction of zeta potential is necessary to help save both time and money. In this study, machine learning methods including MLR, SVM, and ANNs were tested to estimate zeta potential based on various affecting parameters. The following conclusions are made:

- The correlation coefficient for the developed MLR model was found to be 0.68, possibly due to the model’s consideration of seven predictors and the intricate relationship between these predictors and the response variable. This suggests that the model’s ability to predict zeta potential is limited due to the nonlinearity of data.
- The SVM model shows a better correlation between predicted and experimental zeta potential compared to the MLR model, with an excellent correlation coefficient of 0.997 for the training set. This is because the SVM model can capture the nonlinearity of the relationship between predictors and response variables. However, it failed to provide acceptable results for the test data and predictions due to the complexity of the system with several variables.
- Compared to the MLR and SVM models, the ANN model presents the strongest and most significant correlation between predicted and experimental zeta potential for both training and test sets, with correlation coefficients of 0.982 and 0.923, respectively, indicating that the model has a high degree of accuracy. Additionally, the ANN model was able to accurately predict zeta potentials when specific measurement conditions were used as inputs, which were obtained from the literature. The predicted zeta potential closely matched the actual values with correlation coefficients of more than 97%.

## Figures and Tables

**Table 1:** Zeta potential data collection.

Input Parameter	Values	Output	Sources
Silica nanoparticle size	10–50 nm	Zeta potential (mV)	[10,14,52,76,97,98,99,100,101,102,103,104,105,106,107,108,109,110,111,112,113,114,115,116,117]
Nanoparticle concentration	0.005–6 wt%		
Temperature	20–150 °C		
Salinity	NaCl: 30–315,000 ppm KCl: 500–60,000 ppm		
pH	1–11		
Medium	Water; sand; glass beads; coal; kaolinite		

**Table 2:** MLR model performance indexes.

Response Variable	Indices	Value
Zeta potential	Correlation coefficient	0.680
	RMSE	10.722
	MAPE	0.267

**Table 3:** SVM model performance indexes.

Response Variable	Indices	Category	Value
Zeta potential	Correlation coefficient	Training	0.997
		Test	0.516
	RMSE	Training	1.155
		Test	12.271
	MAPE	Training	0.019
		Test	0.288

**Table 4:** ANN model performance indexes.

Response Variable	Indices	Category	Value
Zeta potential	Correlation coefficient	Training	0.982
		Test	0.923
	RMSE	Training	2.754
		Test	5.347
	MAPE	Training	0.053
		Test	0.131

## Data Availability

The data presented in this study are available on request from the corresponding author.

## Supplementary Materials

The following supporting information can be downloaded at: https://www.mdpi.com/article/10.3390/nano13071209/s1, Table S1: Input Data for machine learning models. Reference [124] is cited in the supplementary materials.

## Nomenclature

r	correlation coefficient
x_i_	values of the x-variable in a sample
$\bar{x}$	mean of the values of the x-variable
y_i_	values of the y-variable in a sample
$\bar{y}$	mean of the values of the y-variable
F(*ka*)	Debye function
U_E_	electrophoretic mobility
η	viscosity of the medium
ε	dielectric constant
