# Supplementary material for: Unlocking the Power of Artificial Intelligence: Accurate Zeta Potential Prediction Using Machine Learning
Source: Nanomaterials (Basel). 2023 Mar 29;13(7):1209. doi: 10.3390/nano13071209 (PMC10096557; doi:10.3390/nano13071209)
Supplement: Supplementary file 1 [file nanomaterials-13-01209-s001.zip › nanomaterials-2273616-supplementary.pdf]

Unlocking the Power of Artificial Intelligence: Accurate Zeta Potential Prediction using Machine Learning

Rizwan Muneer [1], Muhammad Rehan Hashmet [2,*], Peyman Pourafshary [1] and Mariam Shakeel [1]

[1] School of Mining and Geosciences, Nazarbayev University, Astana 010000, Kazakhstan.

[2] Department of Chemical and Petroleum Engineering, United Arab Emirates University.

* Corresponding Author

Table S1. Input Data for machine learning models.

Study	*Temp.*	*NPs Size*	*KCl Conc.*	*NaCl Conc.*	*NPs Conc.*	*pH*	*Medium*	*Zeta Potential*
	°C	nm	ppm	ppm	wt%			mV
Parizad et al. [1]	30	15	10000	0	0.1	9.5	Water	-42
	30	15	10000	0	0.25	9.5	Water	-45
	30	15	10000	0	0.75	9.5	Water	-46
	30	15	10000	0	0.25	9.5	Water	-45.1
	50	15	10000	0	0.25	9.5	Water	-43
	70	15	10000	0	0.25	9.5	Water	-39.1
	30	15	10000	0	0.25	9.5	Water	-45.1
	30	15	30000	0	0.25	9.5	Water	-28.1
	30	15	60000	0	0.25	9.5	Water	-15.1
Kun Ma et al. [2]	25	15	0	0	0.05	1	Water	0.342
	25	15	0	0	0.05	2	Water	-1.44
	25	15	0	0	0.05	3	Water	-12.5
	25	15	0	0	0.05	4	Water	-20.1
	25	15	0	0	0.05	5	Water	-25.4
	25	15	0	0	0.05	6	Water	-28.7
	25	15	0	0	0.05	7	Water	-29
	25	15	0	0	0.05	8	Water	-32.1
	25	15	0	0	0.05	9	Water	-20.5
	25	15	0	0	1	7	Water	-42.9
	90	15	0	0	1	7	Water	-40.4
Wang et al. [3]	5	20	0	0	1	7	Water	-39.8
	5	20	0	0	2	7	Water	-36.6
	5	20	0	0	3	7	Water	-31.4
	5	20	0	0	4	7	Water	-25.9
	5	20	0	0	5	7	Water	-24.1
	5	20	0	0	6	7	Water	-22.2
	5	50	0	0	1	7	Water	-47.2
	5	50	0	0	2	7	Water	-42.2

	5	50	0	0	3	7	Water	-35.1
	5	50	0	0	4	7	Water	-27.3
	5	50	0	0	5	7	Water	-25.3
	5	50	0	0	6	7	Water	-24
Bayat et al. [4]	26	20	0	0	0.005	6.4	Water	-28.1
Al-Ansari et al. [5]	25	10	0	10000	0.1	6.3	Water	-5
	25	0	0	10000	0	6.3	Water	-40
Muneer et al. [6]	25	20	0	5844	0.05	7	Water	-28.7
	25	20	0	11688	0.05	7	Water	-21.8
	25	20	0	17532	0.05	7	Water	-18.3
	25	20	0	23376	0.05	7	Water	-15.9
Chequer et al. [7]	22	0	0	35064	0	7	Sand	-17.9
	22	0	0	23376	0	7	Sand	-21.2
	22	0	0	11688	0	7	Sand	-24.2
	22	0	0	5844	0	7	Sand	-30.7
	22	0	0	2922	0	7	Sand	-33.8
	22	0	0	1753.2	0	7	Sand	-34
Fogden et al. [8]	25	0	0	999.3	0	7	Sand	-39
Lebedeva et al. [9]	25	0	0	4383	0	7	Sand	-36.6
	25	0	0	43830	0	7	Sand	-14.7
Hussain et al. [10]	25	0	0	2922	0	7	Sand	-30
	25	0	0	14610	0	7	Sand	-30
	25	0	0	29220	0	7	Sand	-20
Xie et al. [11]	25	0	0	9999.1	0	7	Sand	-30
Shehata et al. [12]	25	0	0	50007.1	0	7	Sand	-12
	25	0	0	5844	0	7	Sand	-31
	25	0	0	5002.5	0	7	Sand	-32
	25	0	0	1987.0	0	7	Sand	-35
Walker et al. [13]	25	0	0	29220	0	7	Sand	-21
	25	0	0	40908	0	7	Sand	-16
Chequer et al. [7]	25	0	0	1461	0	7	Sand	-34
	25	0	0	2922	0	7	Sand	-34
	25	0	0	11688	0	7	Sand	-24
	25	0	0	23376	0	7	Sand	-21
Nasralla et al. [14]	25	0	0	2000	0	7.7	Sand	-35
Xie et al. [15]	65	0	0	2000	0	8.1	Sand	-23.7
Xie et al. [11]	25	0	0	2000	0	8.1	Sand	-33
Yao et al. [16]	25	0	0	0	0	9.2	Sand	-23
Muneer et al. [17]	25	0	0	11688	0	6.9	Sand	-29.5
	25	0	0	8766	0	6.9	Sand	-30.6
	25	0	0	6428.4	0	6.9	Sand	-30.9

	25	0	0	5844	0	6.9	Sand	-30.8
Alkafeef et al. [18]	25	0	0.7	0	0	6	Sand	-75
	25	0	521.9	0	0	6	Sand	-57
	25	0	820.1	0	0	6	Sand	-48
	25	0	2012.9	0	0	6	Sand	-27
	25	0	5964.1	0	0	6	Sand	-16
	25	0	7455.1	0	0	6	Sand	-11
Muneer et al. [6]	24.15	0	0	11688	0	7	Sand	-29.5
	24.15	0	0	8766	0	7	Sand	-30.6
	24.15	0	0	6428.4	0	7	Sand	-30.9
	24.15	0	0	5844	0	7	Sand	-30.8
	24.15	0	0	5259.6	0	7	Sand	-31.6
	24.15	0	0	4967.4	0	7	Sand	-32
	24.15	0	0	4675.2	0	7	Sand	-32.5
	24.15	0	0	4383	0	7	Sand	-33.3
	24.15	0	0	4090.8	0	7	Sand	-32.8
	24.15	0	0	3506.4	0	7	Sand	-33.6
	24.15	0	0	2922	0	7	Sand	-35.2
	24.15	20	0	5844	0.05	7	Sand	-28.2
	24.15	20	0	4967.4	0.05	7	Sand	-28.9
	24.15	20	0	4090.8	0.05	7	Sand	-29.1
	24.15	20	0	2922	0.05	7	Sand	-30.5
	24.15	20	0	5844	0.1	7	Sand	-27
	24.15	20	0	4967.4	0.1	7	Sand	-27.5
	24.15	20	0	3506.4	0.1	7	Sand	-28.5
	24.15	20	0	2922	0.1	7	Sand	-28.6
Vinogradov et al. [19]	21	0	0	876.6	0	6.5	Sand	-20.3
	21	0	0	26298	0	7.1	Sand	-10.9
	21	0	0	11688	0	7.1	Sand	-10.5
	15	0	0	876.6	0	6.5	Sand	-21.9
	21	0	0	876.6	0	6.5	Sand	-20.6
	38	0	0	876.6	0	6.25	Sand	-17.6
	77.1	0	0	876.6	0	6.1	Sand	-15.3
	118	0	0	876.6	0	5.8	Sand	-13.2
	145	0	0	876.6	0	5.8	Sand	-11
Vinogradov et al. [20]	18	0	0	584.4	0	6.8	Sand	-23.9
	39	0	0	584.4	0	6.45	Sand	-15.6
	78	0	0	584.4	0	5.82	Sand	-11.2
	83	0	0	584.4	0	5.95	Sand	-11.4
	118	0	0	584.4	0	5.95	Sand	-10.6
	122	0	0	584.4	0	5.95	Sand	-10.4
	147	0	0	584.4	0	5.95	Sand	-9.17

Vinogradov et al. [19]	19.5	0	0	26298	0	7.25	Sand	-11.2
	21	0	0	26298	0	7.25	Sand	-11.1
	38	0	0	26298	0	7.25	Sand	-10.8
	78	0	0	26298	0	7.25	Sand	-10.4
	118	0	0	26298	0	7.25	Sand	-10.2
	134	0	0	26298	0	7.25	Sand	-9.08
	19.5	0	0	11688	0	7.25	Sand	-10.8
	20.7	0	0	11688	0	7.25	Sand	-10.5
	62.4	0	0	11688	0	7.25	Sand	-10.2
	64.3	0	0	11688	0	7.25	Sand	-10.7
	110	0	0	11688	0	7.25	Sand	-10.9
	111	0	0	11688	0	7.25	Sand	-10.6
Vinogradov et al. [20]	19.5	0	0	29220	0	7.25	Sand	-9.35
	37.3	0	0	29220	0	7.25	Sand	-10.2
	71	0	0	29220	0	7.25	Sand	-8.67
	112	0	0	29220	0	7.25	Sand	-10.5
	140	0	0	29220	0	7.25	Sand	-10.8
Vinogradov et al. [21]	23	0	0	9.8	0	7	Sand	-52
	23	0	0	16.0	0	7	Sand	-56.2
	23	0	0	31.0	0	7	Sand	-55.7
	23	0	0	114.5	0	7	Sand	-52.3
	23	0	0	736.3	0	7	Sand	-45.1
	23	0	0	1916.8	0	7	Sand	-34.2
	23	0	0	3897.9	0	7	Sand	-26.7
	23	0	0	8649.1	0	7	Sand	-16.7
	23	0	0	15428.2	0	7	Sand	-10.3
	23	0	0	37401.6	0	7	Sand	-20.3
	23	0	0	66621.6	0	7	Sand	-23.7
	23	0	0	141425	0	7	Sand	-19.5
	23	0	0	161294	0	7	Sand	-13.1
	23	0	0	314407	0	7	Sand	-18.6
Chequer et al. [7]	22	0	0	35064	0	7	Glass beads	-15.9
	22	0	0	23376	0	7	Glass beads	-16.8
	22	0	0	17532	0	7	Glass beads	-17.2
	22	0	0	11688	0	7	Glass beads	-17.7
	22	0	0	5844	0	7	Glass beads	-24.8
	22	0	0	2922	0	7	Glass beads	-36.7
	22	0	0	1753.2	0	7	Glass beads	-47.5
	22	0	0	584.4	0	7	Glass beads	-54.9
	22	0	0	58.44	0	7	Glass beads	-55.5
Ahmadi et al. [22]	25	48	0	0	0.1	7	Glass beads	-22.5
Hasannejad et al. [23]	25	145	0	17532	0.1	7	Glass beads	-25
	25	0	0	17532	0	7	Glass beads	-28

Mansouri et al. [24]	25	15	0	1753.2	0.1	5.9	Glass beads	-7.5
	25	0	0	1753.2	0	5.9	Glass beads	-25
Assef et al. [25]	25	0	0	1168.8	0	6.5	Glass beads	-24.3
	25	0	745.51	0	0	6.5	Glass beads	-27.7
	25	0	0	0	0	6.5	Glass beads	-39.5
	25	0	745.51	0	0	2	Glass beads	1
	25	0	745.51	0	0	2.5	Glass beads	0.429
	25	0	745.51	0	0	3	Glass beads	-0.143
	25	0	745.51	0	0	3.5	Glass beads	-2.43
	25	0	745.51	0	0	4	Glass beads	-3.57
	25	0	745.51	0	0	4.4	Glass beads	-9
	25	0	745.51	0	0	5.2	Glass beads	-13
	25	0	745.51	0	0	5.9	Glass beads	-21.1
	25	0	745.51	0	0	6.25	Glass beads	-25.1
	25	0	745.51	0	0	7	Glass beads	-39.6
	25	0	745.51	0	0	7.5	Glass beads	-47
	25	0	745.51	0	0	8	Glass beads	-58
	25	0	745.51	0	0	8.75	Glass beads	-59
Huang et al. [26]	25	0	20000	0	0	7	Coal-fines	-12.61
Awan et al. [27]	23	0	0	0	0	6	Coal-fines	-7.7
	23	0	0	0	0	6.5	Coal-fines	-22.4
	23	0	0	0	0	7	Coal-fines	-26.4
	23	0	0	0	0	7.5	Coal-fines	-31.2
	23	0	0	0	0	8	Coal-fines	-33.8
	23	0	0	0	0	8.5	Coal-fines	-35.0
	23	0	0	0	0	9	Coal-fines	-38.7
	23	0	0	0	0	9.5	Coal-fines	-37.9
	23	0	0	0	0	10	Coal-fines	-40.5
	23	0	0	0	0	10.5	Coal-fines	-40.4
	23	0	0	0	0	11	Coal-fines	-42.1
	23	0	0	58.4	0	6	Coal-fines	-3.8
	23	0	0	58.4	0	6.5	Coal-fines	-15.8
	23	0	0	58.4	0	7	Coal-fines	-22.4
	23	0	0	58.4	0	7.5	Coal-fines	-31.6
	23	0	0	58.4	0	8	Coal-fines	-35.1
	23	0	0	58.4	0	8.5	Coal-fines	-41.4
	23	0	0	58.4	0	9	Coal-fines	-47.8
	23	0	0	58.4	0	9.5	Coal-fines	-52.2
	23	0	0	58.4	0	10	Coal-fines	-52.3
	23	0	0	58.4	0	10.5	Coal-fines	-55.9
	23	0	0	58.4	0	11	Coal-fines	-58.3
	23	0	0	584.4	0	6	Coal-fines	2.4
	23	0	0	584.4	0	6.5	Coal-fines	1.7
	23	0	0	584.4	0	7	Coal-fines	-8.6

	23	0	0	584.4	0	7.5	Coal-fines	-18.7
	23	0	0	584.4	0	8	Coal-fines	-28.0
	23	0	0	584.4	0	8.5	Coal-fines	-33.8
	23	0	0	584.4	0	9	Coal-fines	-43.6
	23	0	0	584.4	0	9.5	Coal-fines	-47.5
	23	0	0	584.4	0	10	Coal-fines	-53.0
	23	0	0	584.4	0	10.5	Coal-fines	-56.9
	23	0	0	584.4	0	11	Coal-fines	-58.7
	23	0	0	5844	0	6	Coal-fines	6.9
	23	0	0	5844	0	6.5	Coal-fines	4.7
	23	0	0	5844	0	7	Coal-fines	0.6
	23	0	0	5844	0	7.5	Coal-fines	-7.5
	23	0	0	5844	0	8	Coal-fines	-10.1
	23	0	0	5844	0	8.5	Coal-fines	-15.0
	23	0	0	5844	0	9	Coal-fines	-17.7
	23	0	0	5844	0	9.5	Coal-fines	-21.2
	23	0	0	5844	0	10	Coal-fines	-26.9
	23	0	0	5844	0	10.5	Coal-fines	-29.0
	23	0	0	5844	0	11	Coal-fines	-31.1
	23	0	0	17532	0	6	Coal-fines	-5.1
	23	0	0	17532	0	6.5	Coal-fines	-6.2
	23	0	0	17532	0	7	Coal-fines	-8.0
	23	0	0	17532	0	7.5	Coal-fines	-9.8
	23	0	0	17532	0	8	Coal-fines	-12.8
	23	0	0	17532	0	8.5	Coal-fines	-16.2
	23	0	0	17532	0	9	Coal-fines	-16.9
	23	0	0	17532	0	9.5	Coal-fines	-21.3
	23	0	0	17532	0	10	Coal-fines	-25.2
	23	0	0	17532	0	10.5	Coal-fines	-26.8
	23	0	0	17532	0	11	Coal-fines	-30.1
	23	0	0	35064	0	6	Coal-fines	-6.0
	23	0	0	35064	0	6.5	Coal-fines	-9.5
	23	0	0	35064	0	7	Coal-fines	-13.2
	23	0	0	35064	0	7.5	Coal-fines	-13.7
	23	0	0	35064	0	8	Coal-fines	-15.1
	23	0	0	35064	0	8.5	Coal-fines	-15.9
	23	0	0	35064	0	9	Coal-fines	-17.4
	23	0	0	35064	0	9.5	Coal-fines	-20.4
	23	0	0	35064	0	10	Coal-fines	-21.5
	23	0	0	35064	0	10.5	Coal-fines	-22.9
	23	0	0	35064	0	11	Coal-fines	-27.5
Chequer et al. [7]	22	0	0	35064	0	7	Kaolinite	-23.02
	22	0	0	23376	0	7	Kaolinite	-28.94
	22	0	0	11688	0	7	Kaolinite	-30.37
	22	0	0	5844	0	7	Kaolinite	-32.36

	22	0	0	2922	0	7	Kaolinite	-37.64
	22	0	0	514.3	0	7	Kaolinite	-49.56
Tangparitkul et al. [28]	25	0	0	30.0	0	7	Kaolinite	-50

References

1. Parizad, A.; Shahbazi, K.; Tanha, A.A. SiO2 Nanoparticle and KCl Salt Effects on Filtration and Thixotropical Behavior of Polymeric Water Based Drilling Fluid: With Zeta Potential and Size Analysis. *Results Phys.* **2018**, *9*, 1656–1665.

2. Ma, X.; Lee, N.-H.; Oh, H.-J.; Kim, J.-W.; Rhee, C.-K.; Park, K.-S.; Kim, S.-J. Surface Modification and Characterization of Highly Dispersed Silica Nanoparticles by a Cationic Surfactant. *Colloids Surfaces A Physicochem. Eng. Asp.* **2010**, *358*, 172–176.

3. Wang, R.; Liu, T.; Ning, F.; Ou, W.; Zhang, L.; Wang, Z.; Peng, L.; Sun, J.; Liu, Z.; Li, T. Effect of Hydrophilic Silica Nanoparticles on Hydrate Formation: Insight from the Experimental Study. *J. Energy Chem.* **2019**, *30*, 90–100.

4. Esfandyari Bayat, A.; Junin, R.; Shamshirband, S.; Tong Chong, W. Transport and Retention of Engineered Al2O3, TiO2 and SiO2 Nanoparticles through Various Sedimentary Rocks. *Sci. Rep.* **2015**, *5*, 1–12.

5. Al-Anssari, S.; Arif, M.; Wang, S.; Barifcani, A.; Iglauer, S. Stabilising Nanofluids in Saline Environments. *J. Colloid Interface Sci.* **2017**, *508*, 222–229.

6. Muneer, R.; Hashmet, M.R.; Pourafshary, P. DLVO Modeling to Predict Critical Salt Concentration to Initiate Fines Migration Pre-and Post-Nanofluid Treatment in Sandstones. *SPE J.* **2022**, 1–15.

7. Chequer, L.; Bedrikovetsky, P.; Carageorgos, T.; Badalyan, A.; Gitis, V. Mobilization of Attached Clustered Colloids in Porous Media. *Water Resour. Res.* **2019**, *55*, 5696–5714.

8. Fogden, A. Effect of Water Salinity and PH on the Wettability of a Model Substrate. *Energy & fuels* **2011**, *25*, 5113–5125.

9. Lebedeva, E. V; Fogden, A. Micro-CT and Wettability Analysis of Oil Recovery from Sand Packs and the Effect of Waterflood Salinity and Kaolinite. *Energy & Fuels* **2011**, *25*, 5683–5694.

10. Hussain, F.; Zeinijahromi, A.; Bedrikovetsky, P.; Badalyan, A.; Carageorgos, T.; Cinar, Y. An Experimental Study of Improved Oil Recovery through Fines-Assisted Waterflooding. *J. Pet. Sci. Eng.* **2013**, *109*, 187–197.

11. Xie, Q.; Saeedi, A.; Pooryousefy, E.; Liu, Y. Extended DLVO-Based Estimates of Surface Force in Low Salinity Water Flooding. *J. Mol. Liq.* **2016**, *221*, 658–665.

12. Shehata, A.M.; Nasr-El-Din, H.A. Zeta Potential Measurements: Impact of Salinity on Sandstone Minerals. *Proc. - SPE Int. Symp. Oilf. Chem.* **2015**, *2*, 789–805, doi:10.2118/173763-MS.

13. Walker, E.; Glover, P.W.J. Measurements of the Relationship between Microstructure, PH, and the Streaming and Zeta Potentials of Sandstones. *Transp. porous media* **2018**, *121*, 183–206.

14. Nasralla, R.A.; Nasr-El-Din, H.A. Double-Layer Expansion: Is It a Primary Mechanism of Improved Oil Recovery by Low-Salinity Waterflooding? *Proc. - SPE Symp. Improv. Oil*

Recover. **2012**, *2*, 1624–1640, doi:10.2118/154334-MS.

15. Xie, Q.; Liu, Y.; Wu, J.; Liu, Q. Ions Tuning Water Flooding Experiments and Interpretation by Thermodynamics of Wettability. *J. Pet. Sci. Eng.* **2014**, *124*, 350–358.

16. Yao, J.; Han, H.; Hou, Y.; Gong, E.; Yin, W. A Method of Calculating the Interaction Energy between Particles in Minerals Flotation. *Math. Probl. Eng.* **2016**, *2016*.

17. Muneer, R.; Hashmet, M.R.; Pourafshary, P. Predicting the Critical Salt Concentrations of Monovalent and Divalent Brines to Initiate Fines Migration Using DLVO Modeling. *J. Mol. Liq.* **2022**, *352*, 118690.

18. Alkafeef, S.F.; Alajmi, A.F. Streaming Potentials and Conductivities of Reservoir Rock Cores in Aqueous and Non-Aqueous Liquids. *Colloids Surfaces A Physicochem. Eng. Asp.* **2006**, *289*, 141–148.

19. Vinogradov, J.; Jackson, M.D.; Chamerois, M. Zeta Potential in Sandpacks: Effect of Temperature, Electrolyte PH, Ionic Strength and Divalent Cations. *Colloids Surfaces A Physicochem. Eng. Asp.* **2018**, *553*, 259–271.

20. Vinogradov, J.; Jackson, M.D. Zeta Potential in Intact Natural Sandstones at Elevated Temperatures. *Geophys. Res. Lett.* **2015**, *42*, 6287–6294.

21. Vinogradov, J.; Jaafar, M.Z.; Jackson, M.D. Measurement of Streaming Potential Coupling Coefficient in Sandstones Saturated with Natural and Artificial Brines at High Salinity. *J. Geophys. Res. Solid Earth* **2010**, *115*.

22. Ahmadi, M.; Habibi, A.; Pourafshary, P.; Ayatollahi, S. An Experimental Study of Interaction between Nanoparticles' Deposition on a Sintered Porous Medium and Migratory Fines. *J. Porous Media* **2013**, *16*.

23. HASANNEJAD, R.; Pourafshary, P.; Vatani, A.; Sameni, A. Application of Silica Nanofluid to Control Initiation of Fines Migration. *Pet. Explor. Dev.* **2017**, *44*, 850–859.

24. Mansouri, M.; Nakhaee, A.; Pourafshary, P. Effect of SiO2 Nanoparticles on Fines Stabilization during Low Salinity Water Flooding in Sandstones. *J. Pet. Sci. Eng.* **2019**, *174*, 637–648.

25. Assef, Y.; Arab, D.; Pourafshary, P. Application of Nanofluid to Control Fines Migration to Improve the Performance of Low Salinity Water Flooding and Alkaline Flooding. *J. Pet. Sci. Eng.* **2014**, *124*, 331–340.

26. Huang, F.; Kang, Y.; You, L.; Li, X.; You, Z. Massive Fines Detachment Induced by Moving Gas-Water Interfaces during Early Stage Two-Phase Flow in Coalbed Methane Reservoirs. *Fuel* **2018**, *222*, 193–206.

27. Awan, F.U.R.; Keshavarz, A.; Akhondzadeh, H.; Al-Anssari, S.; Al-Yaseri, A.; Nosrati, A.; Ali, M.; Iglauer, S. Stable Dispersion of Coal Fines during Hydraulic Fracturing Flowback in Coal Seam Gas Reservoirs—an Experimental Study. *Energy & Fuels* **2020**, *34*, 5566–5577.

28. Tangparitkul, S.; Saul, A.; Leelasukseree, C.; Yusuf, M.; Kalantariasl, A. Fines Migration

and Permeability Decline during Reservoir Depletion Coupled with Clay Swelling Due to Low-Salinity Water Injection: An Analytical Study. *J. Pet. Sci. Eng.* **2020**, *194*, 107448.
